# A Multicenter Study to Assess *EGFR* Mutational Status in Plasma: Focus on an Optimized Workflow for Liquid Biopsy in a Clinical Setting

**DOI:** 10.3390/cancers10090290

**Published:** 2018-08-27

**Authors:** Laure Sorber, Karen Zwaenepoel, Koen De Winne, Kaat Van Casteren, Elien Augustus, Julie Jacobs, Xiang Hua Zhang, Daniëlla Galdermans, Els De Droogh, Anneke Lefebure, Ann-Marie Morel, Erika Saenen, Frédérique Bustin, Ingel Demedts, Ulrike Himpe, Thierry Pieters, Paul Germonpré, Sofie Derijcke, Koen Deschepper, Jan P. Van Meerbeeck, Christian Rolfo, Patrick Pauwels

**Affiliations:** 1 Center for Oncological Research Antwerp (CORE), University of Antwerp, 2610 Wilrijk, Belgium; Karen.Zwaenepoel@uza.be (K.Z.); Kaat.VanCasteren@kuleuven.be (K.V.C.); Elien.Augustus@uantwerpen.be (E.A.); Julie.Jacobs@uantwerpen.be (J.J.); Jan.VanMeerbeeck@uza.be (J.P.V.M.); Christian.Rolfo@umm.edu (C.R.); Patrick.Pauwels@uza.be (P.P.); 2 Laboratory of Pathological Anatomy, Antwerp University Hospital (UZA), 2650 Edegem, Belgium; Koen.DeWinne@uza.be; 3 Biomedical Quality Assurance Research Unit, KU Leuven, 3000 Leuven, Belgium; 4 Department of Pulmonology & Thoracic Oncology, Antwerp University Hospital (UZA), 2650 Edegem, Belgium; XiangHua.Zhang@uza.be; 5 Pneumology, ZNA Middelheim, 2020 Antwerp, Belgium; Danny.Galdermans@zna.be (D.G.); Els.DeDroogh@zna.be (E.D.D.); 6 Thoracic Oncology Group Antwerp (TOGA), University of Antwerp, 2610 Wilrijk, Belgium; Anneke.Lefebure@zna.be (A.L.); dr.morel@sjk.be (A.-M.M.); Esaenen@hfr.be (E.S.); Paul.Germonpre@azmmsj.be (P.G.); Koen.Deschepper@aznikolaas.be (K.D.); 7 Pneumology, ZNA STER, 2140 Antwerp, Belgium; 8 Pneumology, AZ Sint-Jozef, 2880 Bornem, Belgium; 9 Lung diseases/Allergology, AZ Heilige Familie, 2840 Reet, Belgium; 10 Pneumology/Allergology, CHR de la Citadelle, 4000 Liège, Belgium; Frederique.Bustin@chrcitadelle.be; 11 Department of Pulmonary diseases, AZ Delta, 8800 Roeselare, Belgium; Ingel.Demedts@azdelta.be (I.D.); Ulrike.Himpe@azdelta.be (U.H.); 12 Pneumology, Cliniques Universitaires Saint-Luc, 1200 Bruxelles, Belgium; Thierry.Pieters@uclouvain.be; 13 Pneumology, AZ Maria Middelares, 9000 Gent, Belgium; 14 Pneumology-Thoracic Oncology, AZ Groeninge, 8500 Kortrijk, Belgium; Sofie.Derijcke@azgroeninge.be; 15 Pneumology, AZ Nikolaas, 9100 Sint-Niklaas, Belgium; 16 Oncology & Phase I Unit-Early Clinical Trials, Antwerp University Hospital (UZA), 2650 Edegem, Belgium; 17 Marlene and Steward Greenebaum Comprehensive Cancer Center, Thoracic Medical Oncology & Early Clinical Trials, Baltimore, MD 12116, USA; 18 Antwerp University Hospital (UZA), Tumor Bank, 2650 Edegem, Belgium

**Keywords:** liquid biopsy, non-small cell lung cancer (NSCLC), *EGFR*, ctDNA, ddPCR

## Abstract

A multicenter study was performed to determine an optimal workflow for liquid biopsy in a clinical setting. In total, 549 plasma samples from 234 non-small cell lung cancer (NSCLC) patients were collected. Epidermal Growth Factor Receptor (*EGFR)* circulating cell-free tumor DNA (ctDNA) mutational analysis was performed using digital droplet PCR (ddPCR). The influence of (pre-) analytical variables on ctDNA analysis was investigated. Sensitivity of ctDNA analysis was influenced by an interplay between increased plasma volume (*p* < 0.001) and short transit time (*p* = 0.018). Multistep, high-speed centrifugation both increased plasma generation (*p* < 0.001) and reduced genomic DNA (gDNA) contamination. Longer transit time increased the risk of hemolysis (*p* < 0.001) and low temperatures were shown to have a negative effect. Metastatic sites were found to be strongly associated with ctDNA detection (*p* < 0.001), as well as allele frequency (*p* = 0.034). Activating mutations were detected in a higher concentration and allele frequency compared to the T790M mutation (*p* = 0.003, and *p* = 0.002, respectively). Optimization of (pre-) analytical variables is key to successful ctDNA analysis. Sufficient plasma volumes without hemolysis or gDNA contamination can be achieved by using multistep, high-speed centrifugation, coupled with short transit time and temperature regulation. Metastatic site location influenced ctDNA detection. Finally, ctDNA levels might have further value in detecting resistance mechanisms.

## 1. Introduction

Non-small cell lung cancer (NSCLC), which makes up approximately 80% of all lung cancer cases, remains one of the world’s leading causes of cancer-related mortality [1]. The latest American Society of Clinical Oncology (ASCO) guidelines concerning systemic therapy for stage IV NSCLC highlight that tumors should be examined for the presence of targetable molecular alterations [2]. Detection of activating Epidermal Growth Factor Receptor (*EGFR)* mutations warrants treatment with EGFR tyrosine kinase inhibitors (TKIs), such as afatinib, erlotinib, or gefitinib). Despite high response rates and durable responses, most patients develop resistance after a median period of 12 months [3]. The *EGFR* T790M mutation accounts for approximately half of the instances in acquired resistance to first line EGFR TKI therapy [4], in which case osimertinib (third generation EGFR TKI) is recommended [2]. Hence, genotyping is important in order to provide patients with the most optimal treatment. Although tissue biopsies remain the gold standard, it is associated with several limitations. Taking a biopsy is an invasive procedure, which can be potentially harmful for the patient. In addition, the tumor cell content can be very low. Liquid biopsy, consisting of circulating cell-free (tumor) DNA (cfDNA/ctDNA) analysis, has emerged as viable approach to guide therapeutic decisions and provide real-time follow-up [5,6]. The development of both targeted, digital-PCR based and (non-) targeted, genome-wide analyses have increased the potential of ctDNA analysis. Several studies have demonstrated high concordance of mutational profiles between tissue, plasma, and most recently urine samples [7,8]. Importantly, detection of *EGFR* mutations in a liquid biopsy is sufficient proof to warrant EGFR TKI therapy [7,9,10,11]. Patients have been found to display similar clinical response to EGFR TKI therapy, irrespective of whether *EGFR* mutations were identified in plasma, urine, or tissue samples. The group of Sacher et al. demonstrated the feasibility of plasma genotyping using the digital droplet PCR (ddPCR), which is a high-throughput technology with a high level of sensitivity and specificity [12]. This study also reported a shorter turnaround time (TAT) in comparison to tissue biopsies, making it a promising approach for guiding precision medicine. In 2016, the cobas^®^ EGFR Mutation Test v2 (Roche Molecular Systems, Inc., Branchburg, NJ, USA) was the first liquid biopsy test to acquire U S Food and Drug Administration (FDA) approval, which was a major advance in the ctDNA liquid biopsy field [10]. At present, it remains the only FDA approved ctDNA analysis test, though more companion diagnostic liquid biopsies are being developed and validated [13].

Liquid biopsy has great potential for molecular pathology in cancer, but standard operating procedures have to be established in order to facilitate broad clinical implementation [6]. In this multicenter study, we have investigated various (pre-) analytical variables to provide an optimized workflow for implementation of liquid biopsy in a clinical setting.

## 2. Results

### 2.1. Patient Cohort

In total, 234 NSCLC patients were included (Table 1), of which 549 plasma samples were collected. These include 77 (32.91%) patients classified as *EGFR* wild type (WT) according to tissue analysis and 51 patients (21.79%) without any information regarding *EGFR* mutational status. *EGFR* mutations were detected in plasma samples of 1 (1.30%) and 3 (5.88%) patients, respectively. The 110 *EGFR* mutated patients (47.01%) were identified by tissue and/or plasma analysis. Of these patients, 38 provided one plasma sample (34.55%) and 68 (61.82%) provided on average six follow-up plasma samples. 101 patients received targeted therapy before or during the study. The *EGFR* T790M resistance mutation was detected in tissue and/or plasma of 34 (30.91%) *EGFR* mutated patients, of which 19 arose during follow-up. In the other patients, the presence of the T790M mutation was already established or suspected at inclusion.

### 2.2. Sample Characteristics

The plasma samples (*n* = 549) were collected primarily in Streck tubes (472, 85.98%). In some cases, K2 Ethylenediaminetetraacetic acid (EDTA) tubes (77, 14.03%) were used (at the start of the multicenter study and in-house inclusion). All samples were grouped based upon the reached assay sensitivity, which is the maximal detectable *EGFR* mutation frequency; non-informative ([>3%] 45, 8.20%), restricted sensitivity ([0.5–3%] 217, 39.52%), and adequate sensitivity ([<0.5%] 287, 52.28%). In total, 106 (19, 3%) samples were hemolytic upon plasma generation. During the study, the centrifugation protocol for Streck tubes (*n* = 472) was altered, with 369 (78.18%) samples processed using the original protocol and 103 (21.82%) with the two-step protocol (Table S1).

### 2.3. Pre-Analytical Variables

Reached assay sensitivity, which is calculated based on total cfDNA input, was significantly influenced by the amount of generated plasma (*p* < 0.001), irrespective of the type of collection tube. In addition, a negative correlation with the presence of hemolysis was observed (correlation coefficient (R) = −0.136, *p* = 0.001). Most samples were collected using Streck tubes. Based on the data obtained from samples collected in these tubes, the effect of several pre-analytical variables (centrifugation protocol, transit time, and ambient temperature (average temperature per month) (Table S1)) on reached assay sensitivity was investigated.

#### 2.3.1. Effect of Pre-Analytical Variables on Plasma Volume and Hemolysis

The two-step centrifugation protocol resulted in a significant increase in the amount of generated plasma (*p* < 0.001). A longer transit time was associated with a lower amount of plasma in the original centrifugation protocol (*p* = 0.005). This effect was not observed in the two-step protocol. Average temperature did not seem to influence plasma generation. Hemolysis was found to be significantly correlated with a lower amount of plasma (*p* < 0.001). Hence, the effect of the pre-analytical variables (centrifugation protocol, transit time and temperature) on the occurrence of hemolysis was also determined. Transit time was the only factor to have a significant impact (*p* < 0.001). In conclusion, transit time had a significant impact on plasma generation and the occurrence of hemolysis. However, this effect on plasma generation could be negated by using a two-step, high-speed centrifugation protocol.

#### 2.3.2. Effect of Pre-Analytical Variables on Reached Assay Sensitivity

Multivariate analysis was performed to determine the effect of the aforementioned pre-analytical variables on reached assay sensitivity (maximal detectable *EGFR* mutation frequency). Centrifugation protocol was significantly associated with reached assay sensitivity (*p* < 0.001). The original protocol resulted in a seemingly higher sensitivity than the two-step protocol, as additional long fragment DNA is removed in case of the latter. An increase in transit time in samples processed using the original protocol was found to negatively influence reached assay sensitivity (*p* = 0.001), whereas the opposite effect was observed in samples from the two-step protocol (*p* = 0.016). A trend was observed towards decreased sensitivity when hemolysis occurred (*p* = 0.047). In conclusion, sensitivity is influenced by the interplay between centrifugation protocol, amount of plasma, and transit time (*p* < 0.001).

The average temperature was found to have no significant effect on either plasma generation or reached assay sensitivity. Interestingly, when looking at the samples processed using the original centrifugation protocol, a significant difference in average temperature per reached assay sensitivity was observed (*p* = 0.005). An average temperature of 8 °C was recorded for samples which generated a non-informative result, while 11.5 (*p* = 0.004) and 10.8 °C (*p* = 0.009) was recorded for samples with restricted and adequate sensitivity, respectively.

## 3. cfDNA Analysis

### 3.1. CtDNA Detection

In this study, the *EGFR* mutated patient cohort (*n* = 110) provided 416 plasma samples, of which 50 samples were taken prior to therapy, 69 at radiologic progression and 297 during follow-up. Mutated ctDNA was detected in 34 (68%) baseline samples and 51 (73.91%) samples at radiologic progression. Detectable levels of mutated ctDNA seemed to be more often found in samples with adequate sensitivity (*EGFR* mutation can be detected at frequencies below 0.5% of the total cfDNA), higher amount of generated plasma and shorter transit time (Table 2). However, no statistical differences between mutated and WT samples were observed with regards to these pre-analytical variables. In 63.16% of the samples with detectable mutated ctDNA, the sample was classified as adequate sensitivity.

The detection of ctDNA was found to be significantly associated with the presence of extrathoracic metastases both prior to treatment (*p* < 0.001) and at radiologic progression (*p* < 0.001). This association was also seen in the absence (*p* = 0.001, and *p* = 0.004) and presence (*p* = 0.038, and *p*= 0.006) of brain metastases in these two settings, respectively. The presence of brain metastases did not influence the detection of mutated ctDNA prior to therapy, irrespective of extrathoracic metastases. Unexpectedly, the presence of brain metastases at radiologic progression was found to positively influence the ctDNA detection (*p* = 0.009). However, this association was no longer detected when taking the presence or absence of extrathoracic metastases into account.

### 3.2. ctDNA Characteristics

**Figure 1:**
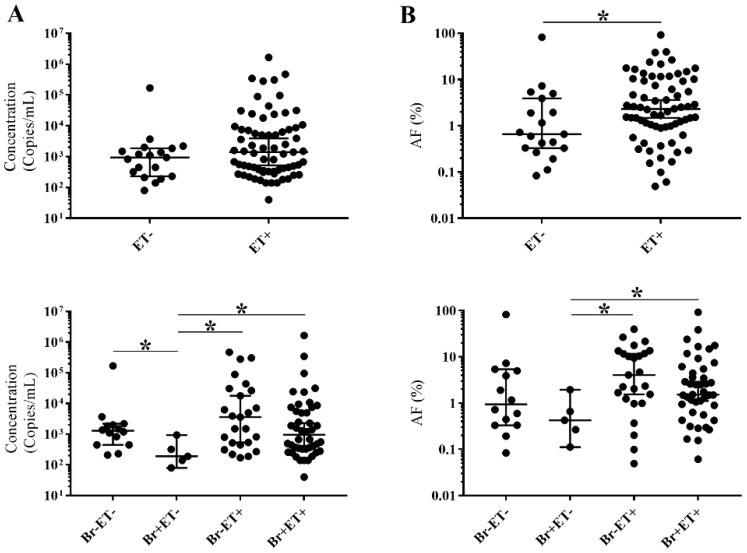
Influence of metastases on detectable *EGFR* mutated ctDNA levels. (**A**) distribution of concentration (copies/mL); (**B**) distribution of allele frequency (%); Br: brain metastases; ET: extrathoracic metastases; AF (%): percentage of allele frequency; +: present; −: absent; median with 95% confidence interval (CI); * *p* < 0.05.

The concentration (copies/mL) and allele frequency (%) of *EGFR* mutated ctDNA (both activating mutations and T790M resistance mutation) in samples taken at radiologic disease progression or prior to therapy were found to be similar in these two groups. Previous treatment schedules also did not seem to influence ctDNA levels. Interestingly, extrathoracic metastases were found to have a significant influence on allele frequency. Furthermore, higher concentrations were detected compared to brain metastases alone (Figure 1).

**Figure 2:**
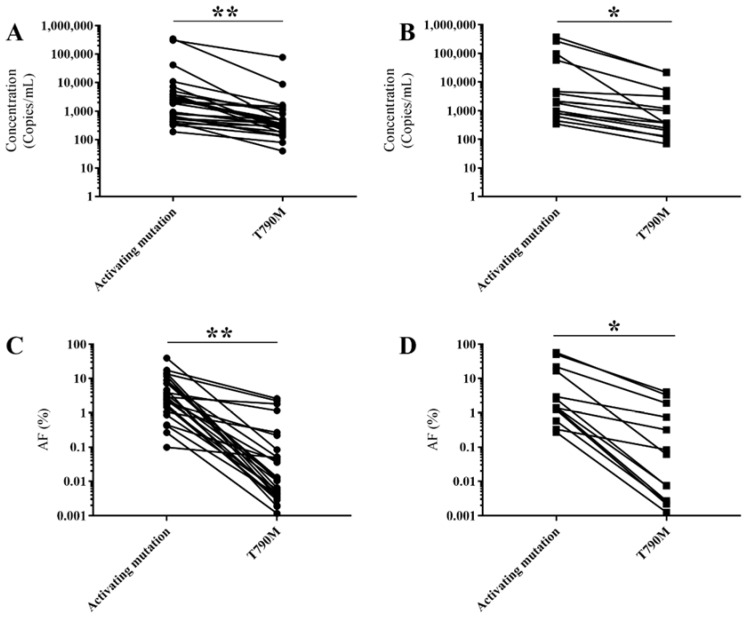
Comparison of ctDNA (activating mutations vs. T790M mutation) (*n* = 43). (**A**,**B**) concentration (copies/mL) of *EGFR* activating mutations (ex19del, L858R, L861Q, and G719X) versus T790M mutation detected in samples taken at radiological progression (*n* = 29) (**A**) and during follow up (*n* = 14) (**B**), respectively. The lowest detectable concentration was 190 and 40 copies/mL, with a median of 1950 and 360 copies/mL, respectively; (**C**,**D**) distribution of the allele frequencies detected in samples taken at radiological progression (*n* = 29) (**C**) and during follow up (*n* = 14), respectively. The lowest allele frequency detected was 0.0985 and 0.0012%, with a median of 2.530 and 0.0107%, respectively; * *p* < 0.05; ** *p* < 0.001.

The dynamics between activating mutations and the T790M mutation were also investigated. The T790M mutation was detected in 29 samples taken at radiologic progression. Both the concentration and allele frequency were lower than those of the activating mutation (*p* < 0.001, and *p* < 0.001, respectively) (Figure 2A,C). Furthermore, the T790M mutation was also detected in 14 follow-up samples without radiologic progression to EGFR TKI therapy, in which a similar distribution was detected (*p* = 0.037, and *p* = 0.002, respectively) (Figure 2B,D). A clear difference in both the concentration and allele frequency was observed between the activating mutation and T790M mutation (*p* < 0.001, and *p* < 0.001, respectively) in 40 out of 43 plasma samples. In three cases, the activating mutation was not detected, mostly due to technical failure of the assay which screens for the activating mutations.

A significant difference in progression free survival (PFS) was detected between patients with or without detectable ctDNA levels (5.8 months vs. 13.3 months; hazard ratio (HR) = 3.646, 95% CI 1.201–11.071; *p* = 0.029) and with or without extrathoracic metastases prior to therapy (8.2 months vs. 14.4 months; HR = 2.082, 95% CI 1.303–3.326; *p* = 0.002). High concentration and allele frequency of the mutated ctDNA at baseline was also found to have an influence on radiologic progression (*p* = 0.004, and *p* = 0.017, respectively)*.* The presence of brain metastases at the start of therapy was not found to affect PFS.

## 4. Discussion

In this study, several (pre-) analytic variables were investigated in order to provide an optimized and standardized workflow to implement liquid biopsy in a clinical setting (Table 3).

Reached assay sensitivity was calculated based on total cfDNA input; however, it is important to note that no distinction could be made between WT ctDNA, normal cfDNA, and genomic DNA (gDNA). The categorization of reached assay sensitivity was based on the required thresholds to detect clinically relevant ctDNA levels, as seen in in-house data and literature [14]. Sensitivity of cfDNA analysis was found to be increased by an interplay between increased plasma volume and short transit time. We hypothesize that the efficacy of Streck tubes is impaired with increased transit time. The main function of these tubes is to stabilize (i) cfDNA and (ii) blood cells and subsequently prevent hemolysis and gDNA contamination. According to Streck, longer storage of blood samples requires an increase of centrifugation speed [15,16], which was also observed in our study. Furthermore, the multistep, high-speed centrifugation not only increased plasma volume, but also decreased the gDNA contamination [17]. This might explain the lower sensitivity in these samples, as well as the seemingly contradictory effect of transit time on reached assay sensitivity per centrifugation protocol. The fact that hemolysis was found to be strongly associated with an increased transit time and coincided with lower plasma volumes supports our hypothesis of impaired function of Streck tubes. Only a slight negative effect on sensitivity was observed, as we did not differentiate between hemolytic samples due to biological or technical causes.

According to the manufacturer’s instructions, Streck tubes remain effective for up to 14 days at 6 °C to 37 °C [16]. However, it has been demonstrated that storage of these tubes at 10 °C or lower was associated with an increase of gDNA as well as hemolysis and lower plasma volumes [18]. Even though we only recorded the average monthly temperature, temperatures below 10 °C were noted to have a detrimental effect on sensitivity in samples processed using the original centrifugation protocol. As the temperature in north western Europe often drops below 10 °C, optimization of transport conditions, such as other types of cfDNA collection tubes or more insulated transport, might aid in the standardization of liquid biopsy in a clinical setting. Further research might reveal specific, favorable temperature intervals by shipping a data logger with each blood sample. In this manner, differences between the outside temperature and the temperature inside the transport box, duration of exposure to aberrant temperatures, etc. can be closely monitored.

The majority of samples in our cohort in which mutated ctDNA was detected were classified as “adequate sensitivity”. This indicates that the characterization is a good indication. Optimization of these pre-analytical variables will ensure the accuracy of our characterization of actual maximum mutated ctDNA detection.

**Figure 3:**
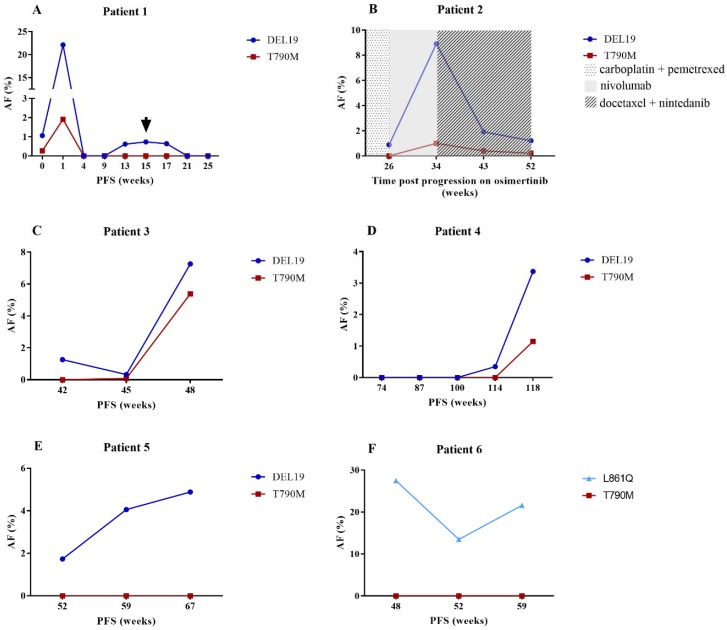
Kinetics of mutated ctDNA. (**A**) initial increase and decrease until undetectable levels of mutated ctDNA after osimertinib therapy. A spike of the activating mutation was detected from week 13 until week 17. At week 15, brain metastases with penetration of the blood brain barrier were detected via brain magnetic resonance imaging (MRI); (**B**) the T790M mutation follows the pattern of the activating mutation throughout several treatment schemes, however, at lower allele frequencies; (**C**,**D**) the activating mutation was detected at very low allele frequencies. A blood sample in a few weeks’ time resulted in the detection of increased allele frequency of this mutation together with the T790M resistance mutation. This corresponded with progressive disease to first- and second-generation EGFR TKI therapy respectively; (**E**,**F**) increasing and extremely high AFs of the activating mutations were detected, respectively. Due to progressive disease under first generation EGFR TKI therapy, tissue biopsies were performed at the last time point, which confirmed the absence of the T790M mutation; AF (%): percentage of allele frequency; PFS: progression-free survival in weeks; 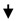: brain MRI.

Pre-analytical variables as well as biological factors have an impact on cfDNA analysis and interpretation. The detection and allele frequency of mutated ctDNA were strongly associated with the presence of extrathoracic metastases. Furthermore, the concentration and allele frequency of mutated ctDNA in these patients were significantly higher than those in patients who only had brain metastases. This is in line with previously reported data in which the relationship between mutated ctDNA detection and both number of metastatic sites and the presence of liver and bone metastases were highlighted [12]. This indicates that tumor load has a significant impact on ctDNA in the circulation and that extrathoracic metastases are responsible for a significant part of the total cfDNA concentration. No effect of brain metastases themselves on the detection, concentration or allele frequency of mutated ctDNA could be discerned. The presence of the blood–brain barrier (BBB) as a physical obstacle could prevent ctDNA from entering the circulation [19]. Only a limited number of patients were included who had brain metastases without an extrathoracic component. Furthermore, it is difficult to detect a negative influence of these brain metastases on the ctDNA concentration in the circulation, especially in the presence of the positive effect of extrathoracic metastases. It is also important to note that information with regards to disruption of the blood brain barrier, or progressive disease due to brain and/or extrathoracic metastases was not available in all cases. However, we speculate that we have been able to detect the moment of BBB penetration in one patient (Figure 3A).

Generally, a higher concentration and allele frequency of the activating mutation was detected compared to the T790M mutation. These findings confirm the data of Oxnard et al. [20], highlighting the importance of testing the majority of the cfDNA isolated from 10 mL of blood in case of T790M ddPCR analysis. In this study, we did not explore whether this difference was due to technical aspects (e.g., assay sensitivities). Furthermore, a recent study reported the correlation of the T790M ratio to the activating mutation and response to osimertinib [21]. Follow-up samples in our study revealed that the T790M mutation closely follows the activating mutation, albeit at a lower concentration and allele frequency (Figure 3B). The activating mutation was also found to be very informative when tracking progressive disease. In a subset of patients, this mutation was detected at very low allele frequency, without the T790M mutation. A blood sample in a few weeks’ time resulted in the detection of both the T790M and activating mutation, the latter in a higher allele frequency (Figure 3C,D). We hypothesize that the T790M mutation was already present in the first sample, but the concentration was too low to detect. Interestingly, increasing and/or high allele frequency of the activating mutation was detected in some patients, without evidence of the T790M mutation (Figure 3E,F). Tissue analysis confirmed absence of the T790M mutation despite the presence of the activating mutation. Further research might determine a clinically relevant threshold in which it is advisable to screen for other resistance mechanisms. In three samples, the T790M mutation was detected without the activating mutation. Presence of the T790M mutation was verified by tissue analysis. In two cases, the activating mutation was missed due to technical issues. To increase the detection of low levels of T790M mutation, it is key to analyze the majority of the cfDNA obtained from 10 mL of blood. Even though there have been reports of T790M detection without the activating mutation [22], most studies highlight coexisting mutations [23,24]. Absence of both mutations was taken as an indication of very low levels of ctDNA in the circulation.

In this study, reached assay sensitivity and detection of the *EGFR* activating mutations were used to determine good quality samples with sufficient ctDNA concentrations. As *EGFR* mutations only represent a fraction of the total ctDNA and are susceptible to EGFR TKI therapy, other biomarkers might be more suitable. Methylated ctDNA (metctDNA) has been shown to be a promising surrogate biomarker of tumor burden in liquid biopsy samples of colorectal cancer patients [25]. Furthermore, metctDNA could function as a prognostic marker, together with mutated ctDNA. The potential prognostic value of mutated ctDNA (concentration) has already been proposed [26,27]. However, discordant results [28] highlight the need for further research to fully optimize liquid biopsy analysis.

## 5. Material and Methods

### 5.1. Study Design

This study was designed as a real-life observational trial of NSCLC patients in several hospitals in Belgium. The objective was to determine the optimal (pre-) analytical settings to assess *EGFR* mutational status using liquid biopsy in a clinical setting. The study was conducted according to the Declaration of Helsinki. Ethical committee approval for this study was obtained from each site with the Ethical committee of the UZA as primary committee (B300201526045, 26 October 2015). All patients provided written informed consent. At inclusion, patients were separated in three groups based on *EGFR* mutational status determined by tissue analysis, namely; (i) tissue analysis was unsuccessful or impossible, (ii) *EGFR* wild type (WT), and (iii) *EGFR* mutated. Patients of the first two groups provided only one blood sample, but, if *EGFR* mutated ctDNA was detected in this sample, they were reassigned to the third group. Multiple blood samples were collected before and during (targeted) therapy and at radiologic disease progression. Feedback with regards to *EGFR* mutation status was provided to the treating thoracic oncologist. Additional information with regards to stage, therapy and the presence or occurrence of brain and extrathoracic metastases was requested.

### 5.2. Sample Collection and Processing

The majority of blood samples were collected using Cell-Free DNA BCT^®^ tubes (Streck tubes; Streck, Biomedical Diagnostics, Antwerp, Belgium). These tubes were shipped to the UZA by post. The number of days from blood collection until arrival at UZA (transit time) and average monthly temperature during transport was recorded. Upon arrival, the blood samples were centrifuged and the generated amount of plasma and presence of hemolysis were documented. In the first part of this study, a one-step, low-speed centrifugation protocol (400× *g* for 10 min) was used. As part of the constant optimization process of the liquid biopsy workflow, this protocol was altered based on in-house comparison [29]. This protocol consists of a two-step, high-speed centrifugation (1600× *g* for 10 min followed by a second step, either 16,000× *g* for 10 min or maximum speed for 1 min). At the start of this study, patients were also included using samples in EDTA tubes. Plasma was generated within 2 h of blood collection, pooled and transported on dry ice. Samples from a small subset of in-house patients were also included using EDTA tubes. Sample collection, processing and storage was performed by Biobank@UZA (Antwerp, Belgium; ID: BE71030031000; Belgian Virtual Tumourbank funded by the National Cancer Plan, BBMRI-ERIC; No. Access: 1, Last: 25 September 2017) [30]. All plasma samples were stored at −80 °C prior to cfDNA isolation.

### 5.3. CfDNA Isolation and Analysis

CfDNA isolation was performed using the Maxwell RSC ccfDNA Large Volume Plasma Kit (Promega, Leiden, The Netherlands) according to the manufacturer’s protocol. The cfDNA was eluted in 65 µL of buffer, irrespective of input plasma volume, ranging from 0.5 to 4 mL. Samples were stored at −20 °C until genotyping. *EGFR* mutational analysis was performed by ddPCR (BioRad, Temse, Belgium). Two multiplex ddPCR assays were designed to detect (i) exon 19 hotspot deletions and the T790M resistance mutation and (ii) the L858R, L861Q and G719A/C/S activating mutations, respectively. The assays were composed of primers and probes described by Oxnard et al. [21] and in-house design (sequences, concentrations, ddPCR mix and thermal cycling conditions are available in Supplementary data). Detailed procedures can be found elsewhere [31]. In case of (suspected) progressive disease, samples were also analyzed using the T790M primer/probe assay from BioRad, according to the manufacturer’s instructions. These samples were analyzed in triplicate to decrease sampling error and increase detection rate. In 2016, these assays and the related workflow received accredited status by BELAC (the Belgian Accreditation Institution). Samples were grouped based on maximal attainable sensitivity (reached assay sensitivity), which is dependent on the previously determined threshold of a positive target event (e.g., T790M) and total cfDNA input. The classification was based on detectable *EGFR* mutation frequencies as seen in in-house data and literature [14]. Hence, samples were categorized as non-informative (>3%), restricted (0.5–3%), or adequate (<0.5%) for the multiplex assays. Thresholds for the T790M assay were lower, namely >1%, 0.1–1%, and <0.1%, respectively. cfDNA isolation, analysis and subsequent feedback to the treating thoracic oncologist were performed once a week. Hence, a TAT of approximately five working days, from sample arrival at UZA until feedback, was generally achieved.

### 5.4. Statistical Analysis

Linear regression was performed to determine the influence of plasma volume on reached assay sensitivity. The effect of pre-analytical variables, including transit time, centrifugation protocol, and average temperature, on plasma generation and the occurrence of hemolysis was investigated using linear and logistic regression, respectively. Next, the impact of all these variables, including plasma volume and hemolysis, on reached assay sensitivity was examined using multivariate analysis. In a subset of samples, one-way ANOVA was performed to determine the effect of temperature on reached assay sensitivity. Whether the presence of brain and/or extrathoracic metastases influenced ctDNA detection was analyzed using chi square analysis, with the Fisher’s exact test to determine the effect of these metastases in relation to each other. Furthermore, the Mann–Whitney U test was performed to investigate their effect on ctDNA concentration and allele frequency, as well as the dynamics between the activating and T790M mutation, ctDNA prior to therapy and at radiologic progression, and lines of treatment. Survival estimates and hazard ratio were calculated according to the Kaplan–Meier method and Cox regression, respectively. ROC curve analysis was used to assess whether ctDNA concentration and allele frequency prior to therapy has an influence on the occurrence of radiologic progression. Statistical analysis was performed using IBM SPSS Statistics version 24.0 (IBM, Brussels, Belgium). *p*-Values of <0.05 were considered statistically significant. Figures were made with Graphpad Prism 7 (Prism 7, Graphpad Software Inc., La Jolla, CA, USA).

## 6. Conclusions

To conclude, optimization of (pre-) analytical variables is key to successful cfDNA analysis. Sufficient plasma volumes without hemolysis or gDNA contamination can be achieved by using multistep, high speed centrifugation coupled with short transit time and precautions in case of low temperatures. Deviating pre-analytical variables, such as low levels of ctDNA in the circulation due to brain and no extrathoracic metastases, might hinder ctDNA detection. In the case of using liquid biopsy as a tool to detect resistance, the detection of low allele frequency of the activating mutation without the T790M resistance mutation might be indicative of low ctDNA levels in the circulation. If clinically possible, a new liquid biopsy in a few weeks might reveal the T790M mutation. A tissue biopsy is advisable in case of either no detectable ctDNA or high allele frequency of the activating mutation alone. The detection of the T790M mutation in the ctDNA is sufficient to initiate osimertinib therapy. Complementary metctDNA analysis, as a biomarker for total ctDNA concentration, might improve real-time follow-up. In this study, an optimized workflow for liquid biopsy has been established which might aid in a further standardization and implementation in clinical practice.

## Figures and Tables

**Table 1:** Overview of patient cohort.

Parameters	*N* (%)
*EGFR* WT (*n* = 124)	
Median age (years)	67
Stage at inclusion	
I	13 (10.48)
II	5 (4.03)
III	26 (20.97)
IV	75 (60.78)
Unspecified	5 (4.03)
*EGFR* mutated (*n* = 110)	
Median age (years)	67.5
Stage at inclusion	
I	4 (3.64)
II	4 (3.64)
III	10 (9.09)
IV	89 (80.91)
Unspecified	3 (2.73)
*EGFR modification as defined by tissue and/or plasma analysis*	
DEL19	63 (57.27)
T790M mutated	26 (41.26)
L858R	32 (29.09)
T790M mutated	6 (18.75)
L861Q	3 (2.73)
T790M mutated	1 (33.33)
G719X	4 (3.64)
T790M mutated	1 (25)
Other	8 (7.27)
Therapy (*n* = 101 *)	
erlotinib	42
PFS (months)	12.3
gefitinib	20
PFS (months)	12.7
afatinib	21
PFS (months)	11.4
osimertinib	16
PFS (months)	8.5
Metastases (at inclusion)	
Brain	
yes	26 (23.64)
no	75 (68.18)
na	9 (8.18)
Extrathoracic	
yes	40 (36.36)
no	61 (55.46)
na	9 (8.18)

*N*: number of patients; Na: not available; PFS: progression-free survival; WT: wild type; *: two patients were included in a study, erlotinib vs. osimertinib in first line, hence no information with regards to their therapy was available.

**Table 2:** Detection of mutations in plasma samples taken prior to therapy and at radiologic progression in relation to pre-analytical variables.

Sampling Time	*EGFR* Mutational Stage	ctDNA Sensitivity Adequate	Plasma Volume mL	Transit Time Days	Average Temperature °C	Centrifugation Protocol Two-Step	Hemolysis Status Present
PtT (50)	WT (16)	9 (56.3%)	3.00	1.77	13.0	5 (38.5%)	3 (18.8%)
	Mutated (34)	21 (61.8%)	3.00	1.59	11.0	10 (34.5%)	7 (20.6%)
PD (69)	WT (21)	12 (60%)	2.85	1.67	11.6	8 (40%)	6 (28.6%)
	Mutated (48)	29 (61.7%)	3.00	1.34	12.5	14 (35.9%)	5 (10.4%)

Centrifugation protocol-original (one-step, low-speed) vs. (two-step, high-speed); ctDNA: circulating, cell-free tumor DNA; adequate sensitivity (<0.5%); PD: progressive disease; PtT: prior to therapy; WT: wild type.

**Table 3:** Optimized workflow for liquid biopsy in a clinical setting.

Variables	Protocol	Specifications
**Pre-analytical variables**	**Streck tubes**	
	Centrifugation protocol	Two-step, high speed: ↑ plasma volume & ↓ gDNA contamination
	Transit time	Short: to ensure proper cell and cfDNA stabilization in Streck tubes
	Temperature	>10 °C: to ensure proper cell and cfDNA stabilization in Streck tubes
	**EDTA tubes**	
	Processing	Within 2 hours: no liquid biopsy-specific preservatives present
	Centrifugation protocol	Two step: ↓ gDNA contamination
**Analytical & biological variables**	**ddPCR**	
	Reached assay sensitivitiy	Indication of cfDNA concentration
	T790M mutation	Test the majority of the isolated cfDNA: lower concentration & AF than activating mutation↑ ctDNA detection
	**Metastases**	
	Extrathoracic	
	Intrathoracic	Very high sensitivity is necessary due to low ctDNA concentrations
	Brain	Disruption of BBB ↑ ctDNA detection
**Interpretation**	**ctDNA detection**	
	No *EGFR* mutation	Tissue biopsy
	*EGFR* activating mutation	
	Prior to therapy	EGFR TKI therapy is recommended
	Without T790M mutation at progressive disease to EGFR TKI therapy	
	Low AF	New blood sample in a few weeks time
	High AF	Tissue biopsy
	*EGFR* (activating &) T790M mutation	Osimertinib therapy is recommended

AF: allele frequency; BBB: blood brain barrier; cfDNA: circulating cell-free DNA; ctDNA: circulating cell-free tumor DNA; gDNA: genomic DNA; ↑: increase; ↓ decrease.

## Supplementary Materials

The following are available online at http://www.mdpi.com/2072-6694/10/9/290/s1, Table S1: Data overview; Description of ddPCR assays (including primer and probe sequences, and mix concentrations).

## References

[1] Jemal A., Bray F., Center M.M., Ferlay J., Ward E., Forman D. (2011). Global cancer statistics. CA Cancer J. Clin..

[2] Hanna N., Johnson D., Temin S., Baker S., Brahmer J., Ellis P.M., Giaccone G., Hesketh P.J., Jaiyesimi I., Leighl N.B. (2017). Systemic therapy for stage iv non-small-cell lung cancer: American society of clinical oncology clinical practice guideline update. J. Clin. Oncol..

[3] Maemondo M., Inoue A., Kobayashi K., Sugawara S., Oizumi S., Isobe H., Gemma A., Harada M., Yoshizawa H., Kinoshita I. (2010). Gefitinib or chemotherapy for non-small-cell lung cancer with mutated egfr. N. Engl. J. Med..

[4] Sequist L.V., Waltman B.A., Dias-Santagata D., Digumarthy S., Turke A.B., Fidias P., Bergethon K., Shaw A.T., Gettinger S., Cosper A.K. (2011). Genotypic and histological evolution of lung cancers acquiring resistance to egfr inhibitors. Sci. Transl. Med..

[5] Sorber L., Zwaenepoel K., Deschoolmeester V., Van Schil P.E., Van Meerbeeck J., Lardon F., Rolfo C., Pauwels P. (2017). Circulating cell-free nucleic acids and platelets as a liquid biopsy in the provision of personalized therapy for lung cancer patients. Lung Cancer.

[6] Stewart C.M., Kothari P.D., Mouliere F., Mair R., Somnay S., Benayed R., Zehir A., Weigelt B., Dawson S.J., Arcila M.E. (2018). The value of cell-free DNA for molecular pathology. J. Pathol..

[7] Siravegna G., Marsoni S., Siena S., Bardelli A. (2017). Integrating liquid biopsies into the management of cancer. Nat. Rev. Clin. Oncol..

[8] Reckamp K.L., Melnikova V.O., Karlovich C., Sequist L.V., Camidge D.R., Wakelee H., Perol M., Oxnard G.R., Kosco K., Croucher P. (2016). A highly sensitive and quantitative test platform for detection of nsclc egfr mutations in urine and plasma. J. Thorac. Oncol..

[9] Goldman J.W., Karlovich C., Sequist L.V., Melnikova V., Franovic A., Gadgeel S.M., Reckamp K.L., Camidge D.R., Pérol M., Ou S.-H.I. (2018). Egfr genotyping of matched urine, plasma, and tumor tissue in patients with non-small-cell lung cancer treated with rociletinib, an egfr tyrosine kinase inhibitor. Precis. Oncol..

[10] Cobas EGFR Mutation Test v2. http://www.Fda.Gov/drugs/informationondrugs/approveddrugs/ucm504540.Htm.

[11] Oxnard G.R., Thress K.S., Alden R.S., Lawrance R., Paweletz C.P., Cantarini M., Yang J.C., Barrett J.C., Janne P.A. (2016). Association between plasma genotyping and outcomes of treatment with osimertinib (azd9291) in advanced non-small-cell lung cancer. J. Clin. Oncol..

[12] Sacher A.G., Paweletz C., Dahlberg S.E., Alden R.S., O’Connell A., Feeney N., Mach S.L., Janne P.A., Oxnard G.R. (2016). Prospective validation of rapid plasma genotyping for the detection of egfr and kras mutations in advanced lung cancer. JAMA Oncol..

[13] Krishnamurthy N., Spencer E., Torkamani A., Nicholson L. (2017). Liquid biopsies for cancer: Coming to a patient near you. J. Clin. Med..

[14] Lee J.Y., Qing X., Xiumin W., Yali B., Chi S., Bak S.H., Lee H.Y., Sun J.M., Lee S.H., Ahn J.S. (2016). Longitudinal monitoring of egfr mutations in plasma predicts outcomes of nsclc patients treated with egfr tkis: Korean lung cancer consortium (klcc-12-02). Oncotarget.

[15] Moore C., Diaz I.M. Controlling Pre-Analytical Variables in Liquid Biopsy Assay Development. http://www.healthtech.com/streck/controlling-pre-analytical-variables/.

[16] Streck Cell-free DNA bct: Instructions for use. https://www.streck.com/collection/cell-free-dna-bct/.

[17] Sherwood J.L., Corcoran C., Brown H., Sharpe A.D., Musilova M., Kohlmann A. (2016). Optimised pre-analytical methods improve kras mutation detection in circulating tumour DNA (ctdna) from patients with non-small cell lung cancer (nsclc). PLoS ONE.

[18] Diaz I.M., Nocon A., Mehnert D.H., Fredebohm J., Diehl F., Holtrup F. (2016). Performance of streck cfdna blood collection tubes for liquid biopsy testing. PloS ONE.

[19] Bettegowda C., Sausen M., Leary R.J., Kinde I., Wang Y., Agrawal N., Bartlett B.R., Wang H., Luber B., Alani R.M. (2014). Detection of circulating tumor DNA in early- and late-stage human malignancies. Sci. Transl. Med..

[20] Oxnard G.R., Paweletz C.P., Kuang Y., Mach S.L., O’Connell A., Messineo M.M., Luke J.J., Butaney M., Kirschmeier P., Jackman D.M. (2014). Noninvasive detection of response and resistance in egfr-mutant lung cancer using quantitative next-generation genotyping of cell-free plasma DNA. Clin. Cancer Res..

[21] Ariyasu R., Nishikawa S., Uchibori K., Oh-Hara T., Yoshizawa T., Dotsu Y., Koyama J., Saiki M., Sonoda T., Kitazono S. (2018). High ratio of t790m to egfr activating mutations correlate with the osimertinib response in non-small-cell lung cancer. Lung Cancer.

[22] Zheng D., Ye X., Zhang M.Z., Sun Y., Wang J.Y., Ni J., Zhang H.P., Zhang L., Luo J., Zhang J. (2016). Plasma egfr t790m ctdna status is associated with clinical outcome in advanced nsclc patients with acquired egfr-tki resistance. Sci. Rep..

[23] Chen H.J., Mok T.S., Chen Z.H., Guo A.L., Zhang X.C., Su J., Wu Y.L. (2009). Clinicopathologic and molecular features of epidermal growth factor receptor t790m mutation and c-met amplification in tyrosine kinase inhibitor-resistant chinese non-small cell lung cancer. Pathol. Oncol. Res..

[24] Tanaka K., Nosaki K., Otsubo K., Azuma K., Sakata S., Ouchi H., Morinaga R., Wataya H., Fujii A., Nakagaki N. (2017). Acquisition of the t790m resistance mutation during afatinib treatment in egfr tyrosine kinase inhibitor-naive patients with non-small cell lung cancer harboring egfr mutations. Oncotarget.

[25] Garrigou S., Perkins G., Garlan F., Normand C., Didelot A., Le Corre D., Peyvandi S., Mulot C., Niarra R., Aucouturier P. (2016). A study of hypermethylated circulating tumor DNA as a universal colorectal cancer biomarker. Clin. Chem..

[26] Zhu Y.J., Zhang H.B., Liu Y.H., Zhang F.L., Zhu Y.Z., Li Y., Bai J.P., Liu L.R., Qu Y.C., Qu X. (2017). Estimation of cell-free circulating egfr mutation concentration predicts outcomes in nsclc patients treated with egfr-tkis. Oncotarget.

[27] Karachaliou N., Mayo-de las Casas C., Queralt C., de Aguirre I., Melloni B., Cardenal F., Garcia-Gomez R., Massuti B., Sanchez J.M., Porta R. (2015). Association of egfr l858r mutation in circulating free DNA with survival in the eurtac trial. JAMA Oncol..

[28] Douillard J.Y., Ostoros G., Cobo M., Ciuleanu T., Cole R., McWalter G., Walker J., Dearden S., Webster A., Milenkova T. (2014). Gefitinib treatment in egfr mutated caucasian nsclc: Circulating-free tumor DNA as a surrogate for determination of egfr status. J. Thorac. Oncol..

[29] Sorber L., Zwaenepoel K., Winne K.D., Jacobs J., Peeters M., Meerbeeck J.V., Rolfo C., Pauwels P. (2018). Two-step, high-seed centrifugation yields higher plasma amount and less genomic DNA contamination.

[30] Goethals S., De Wilde A., Lesage K., Smits E., Pauwels P., Peeters M. (2018). Tumorbank@uza: A collection of tissue, fluid samples and associated data of oncology patients for the use in translational research. Open J. Bioresources.

[31] Sorber L., Zwaenepoel K., Deschoolmeester V., Roeyen G., Lardon F., Rolfo C., Pauwels P. (2017). A comparison of cell-free DNA isolation kits: Isolation and quantification of cell-free DNA in plasma. J. Mol. Diagn..

