# Supplementary material for: A Multicenter Study to Assess EGFR Mutational Status in Plasma: Focus on an Optimized Workflow for Liquid Biopsy in a Clinical Setting
Source: Cancers (Basel). 2018 Aug 27;10(9):290. doi: 10.3390/cancers10090290 (PMC6162772; doi:10.3390/cancers10090290)
Supplement: Supplementary file 1 [file cancers-10-00290-s001.pdf]

Supplementary Materials: A Multicenter Study to Assess EGFR Mutational Status in Plasma: Focus on an Optimized Workflow for Liquid Biopsy in a Clinical Setting

Laure Sorber, Karen Zwaenepoel, Koen De Winne, Kaat Van Casteren, Elien Augustus, Julie Jacobs, Xiang Hua Zhang, Daniëlla Galdermans, Els De Droogh, Anneke Lefebure, Ann-Marie Morel, Erika Saenen, Frédérique Bustin, Ingel Demedts, Ulrike Himpe, Thierry Pieters, Paul Germonpré, Sofie Derijcke, Koen Deschepper, Jan P. Van Meerbeeck, Christian Rolfo, Patrick Pauwels

Multiplex assays: primer and probe sequences, and concentration

EGFR Multiplex 1

Exon 19 deletion hotspot

- Forward primer: 5′-GTGAGAAAGTTAAAATTCCCGTC-3′ – 1.875 μM
- Reverse primer: 5′-CACACAGCAAAGCAGAAAC-3′ – 1.875 μM
- Probe deletion hotspot: 5'-FAM-AGGAATTAAGAGAAGCAACATC-MGB-NFQ-3' – 1 μM
- Probe reference region: 5'-VIC-ATCGAGGATTTCCTTGTTG-MGB-NFQ-3' – 1 μM

Exon 20: T790M mutation

- Forward primer: 5′-GCCTGCTGGGCATCTG-3′ – 0.94 μM
- Reverse primer: 5'-AGCAGGTACTGGGAGCCAATA-3' – 0.94 μM
- Probe: 5′-FAM-ATGAGCTGCATGATGAG-MGB-NFQ-3' – 0.5 μM

EGFR Multiplex 2

Exon 19: G719A/C/S mutations

- Forward primer: 5′- CCTTATACACCGTGCCGAAC-3′ – 1.875 μM
- Reverse primer: 5′- CCTTATACACCGTGCCGAAC-3′ – 1.875 μM
- Probe mutations: 5'-FAM-AGCCCAGCACTTTGATCTTT-MBG-NFQ-3' – 1 μM
- Probe WT: 5'-VIC-CCTCAAGAGAGCTTGGTTGG -MGB-NFQ-3' – 1 μM

Exon 21: L858R mutation

- Forward primer: 5′-GCAGCATGTCAAGATCACAGATT-3′ – 2.85 μM
- Reverse primer: 5′-CCTCCTTCTGCATGGTATTCTTTCT-3′ – 2.85 μM
- Probe mutation: 5'-FAM-AGTTTGGCCCGCCCAA-MGB-NFQ-3' – 1.75 μM

Exon 21: L861Q mutation

- Forward and reverse primer from exon 21 L858R mutation
- Probe mutation: 5'-FAM-CTGGCCAAACAGCTGGGT-MGB-NFQ-3' – 0.75 μM

Digital Droplet PCR (ddPCR)

Both ddPCR multiplex assays were assembled by 10.5 µL 2x ddPCR Supermix for Probes (BioRad), 2 µL primer/probe mix, and 8.5 µL of isolated sample. Assays could be run on the same PCR plate, as they had identical thermal cycling conditions: 95°C for 10 minutes, 40 cycles of 95°C for 30 seconds, then 62°C for 1 minute, followed by 98°C for 10 minutes and cooling to 4°C.

Table S1: Data overview.

Patient	Sample No	EGFR status	Sens	Tube	CP	Transit Time	Hemolysis	#mL	Average Temp	EGFR mut ctDNA
1	1	0	1	0	0	1	0	0.5	16.0	na
2	1	0	0	0	0	6	1	0.5	16.0	na
3	1	0	2	0	0	2	0	3.0	4.7	na
4	1	0	2	0	0	0	0	2.0	17.5	na
5	1	0	1	0	1		0	3.0	13.3	na
6	1	0	2	0	1	5	1	3.0	15.0	na
7	1	0	2	0	0	1	0	2.0	16.0	na
8	1	0	1	0	1	3	0	4.0	13.3	na
9	1	0	2	0	0	1	0	3.0	4.7	na
10	1	0	1	0	1	4	0	3.0	15.0	na
11	1	0	1	0	1	2	0	3.5	6.6	na
12	1	0	2	0	0	0	0	4.0	20.0	na
13	1	0	2	0	0	0	0	3.5	20.0	na
14	1	0	1	0	0	0	0	2.5	8.5	na
15	1	0	2	0	0	1	0	3.0	6.1	na
16	1	0	2	0	0	1	0	4.0	6.1	na
17	1	0	2	0	0	0	0	4.0	15.5	na
18	1	0	2	0	0	0	0	4.0	15.5	na
19	1	0	2	0	1	3	0	3.0	18.1	na
20	1	0	2	0	1	1	0	4.0	4.7	na
20	2	0	1	0	0	3	0	1.5	9.6	na
21	1	0	0	0	0	10	0	1.5	1.1	na
22	1	0	2	0	0	1	0	4.0	1.1	na
23	1	0	1	0	0	4	1	1.0	14.2	na
24	1	0	2	0	0	1	0	2.0	14.2	na
25	1	0	1	0	0	1	0	2.0	1.1	na
26	1	0	1	0	0	1	0	1.5	8.5	na
27	1	0	0	0	0	3	0	0.5	8.5	na
28	1	0	1	0	0	0	0	4.0	18.1	na
29	1	0	2	0	0	3	1	2.0	9.7	na
30	1	0	2	0	0	0	0	4.0	6.1	na
31	1	0	2	0	0	1	1	4.0	1.1	na
32	1	0	2	0	0	3	0	4.5	1.1	na
33	1	0	2	0	0	0	0	3.5	18.3	na
34	1	0	1	0	1	0	1	3.0	13.3	na
35	1	0	0	0	0	5	1	1.0	9.7	na

36	1	0	0	0	0		0	0.5	9.7	na
37	1	0	1	0	0	0	0	4.0	14.2	na
38	1	0	2	0	0	4	0	2.0	9.7	na
39	1	0	0	0	0	8	1	0.5	6.1	na
40	1	0	1	0	0	0	1	1.5	9.6	na
41	1	0	2	0	0	1	0	3.0	14.2	na
42	1	0	2	0	0	0	0	3.5	9.6	na
43	1	0	1	0	0	1	1	1.0	14.2	na
44	1	0	2	0	0	0	0	1.0	16.0	na
45	1	0	1	0	0	0	1	1.0	16.0	na
46	1	0	2	0	0	0	0	3.5	16.0	na
47	1	0	2	0	0	2	1	1.5	16.0	na
48	1	0	0	0	0	0	1	2.0	5.3	na
49	1	0	1	0	0	0	0	4.0	6.1	na
50	1	0	1	0	0	1	0	1.0	5.3	na
51	1	0	0	0	0	0	0	2.0	18.1	na
52	1	0	2	0	0	1	0	3.5	8.5	na
53	1	0	0	0	0	2	1	1.0	18.3	na
54	1	0	2	0	0	1	0	2.5	16.0	na
55	1	0	1	0	0	0	1	3.0	16.0	na
56	1	0	1	0	0	7	1	2.0	6.1	na
57	1	0	1	0	1	1	0	4.0	19.2	na
58	1	0	2	0	0	1	0	4.0	17.5	na
59	1	0	0	0	0	4	0	1.0	6.1	na
60	1	0	2	0	0	1	0	2.0	6.1	na
61	1	0	1	0	0	2	1	1.0	6.1	na
62	1	0	2	0	0	1	0	4.0	4.7	na
63	1	0	2	0	0	1	0	3.0	4.7	na
64	1	0	0	0	0	1	1	0.5	4.7	na
65	1	0	2	0	0	1	1	4.0	4.7	na
66	1	0	2	0	0	1	0	4.0	4.7	na
67	1	0	2	0	0	1	0	4.0	4.7	na
68	1	0	2	0	0	1	0	3.0	1.1	na
69	1	0	2	0	0	1	0	3.5	17.5	na
70	1	0	1	0	0	1	1	4.0	1.1	na
71	1	0	2	0	0	1	0	3.5	1.1	na
72	1	0	2	0	0	1	0	2.0	6.1	na
73	1	0	2	0	0	1	0	2.5	6.1	na
74	1	0	1	0	0	1	1	3.0	6.1	na
75	1	0	1	0	0	1	1	4.0	6.1	na
76	1	0	2	0	0	1	0	4.0	9.6	na
77	1	0	2	0	0	1	0	4.0	9.6	na
78	1	0	2	0	0	1	0	4.0	8.8	na
79	1	0	1	0	0	1	0	4.0	8.8	na
80	1	0	2	0	0	1	0	4.0	9.7	na
81	1	0	2	0	0	1	0	4.0	15.5	na
82	1	0	2	0	0	2	0	4.0	15.5	na

83	1	0	2	0	0	1	0	3.0	15.5	na
84	1	0	2	0	0	1	0	4.0	15.5	na
85	1	0	2	0	0	6	1	3.5	15.5	na
86	1	0	1	0	1	0	0	4.0	19.2	na
87	1	0	1	0	1	1	0	4.0	18.6	na
88	1	0	2	0	1	1	0	4.0	18.6	na
89	1	0	1	0	1	1	1	4.0	18.1	na
90	1	0	2	0	1	1	0	4.0	18.1	na
91	1	0	2	0	0	1	0	4.0	9.7	na
92	1	0	2	0	1	1	0	4.0	18.1	na
93	1	0	1	0	0	1	0	1.0	9.7	na
94	1	0	1	0	0	1	0	1.0	9.7	na
95	1	0	0	0	0	5	0	1.0	6.1	na
96	1	0	2	0	0	4	0	1.5	6.1	na
97	1	0	2	0	0	0	0	3.5	20.0	na
98	1	0	2	0	0	0	0	4.0	20.0	na
98	2	0	2	0	0	0	0	4.0	20.0	na
99	1	0	1	0	0	0	0	4.0	20.0	na
100	1	0	2	0	0	0	0	4.0	20.0	na
101	1	0	2	0	0	0	0	4.0	16.0	na
102	1	0	1	0	0	3	0	3.5	16.0	na
103	1	0	2	0	0	1	0	3.0	18.3	na
103	2	0	2	0	0	2	0	3.0	17.5	na
104	1	0	1	0	0	0	0	3.0	16.0	na
104	2	0	2	0	0	0	0	4.0	17.5	na
105	1	0	2	0	0	0	1	4.0	18.3	na
105	2	0	2	0	0	0	0	4.0	17.5	na
105	3	0	0	0	0	2	1	3.0	1.1	na
106	1	0	1	0	0	0	0	4.0	18.1	na
107	1	0	1	0	0	1	0	2.0	17.5	na
108	1	0	1	0	0	0	0	3.0	8.5	na
109	1	0	2	0	1	6	0	4.0	18.1	na
110	1	0	1	0	0	0	0	4.0	8.5	na
111	1	0	2	0	0	2	1		14.2	na
112	1	0	2	0	0	1	0	2.0	14.2	na
113	1	0	2	0	0	0	0	3.0	16.0	na
114	1	0	1	0	0	3	0	2.0	18.3	na
115	1	0	2	0	0	4	0	1.0	18.1	na
116	1	0	0	0	0	0	0	3.5	1.1	na
117	1	0	0	0	0	0	0	4.0	1.1	na
118	1	0	2	0	0	0	0	3.0	1.1	na
119	1	0	1	0	0	2	0	2.0	4.7	na
120	1	0	2	0	0	3	1	2.5	18.3	na
121	1	0	2	0	0	0	1	4.0	9.7	na
122	1	0	1	0	0	2	1	1.0	1.1	na
123	1	0	2	0	0	1	0	2.0	9.7	na
123	2	0	1	0	0	0	0	3.0	1.1	na

123	3	0	2	0	0		1	3.0	8.8	na
123	4	0	1	0	1	0	0	3.5	18.6	na
124	1	0	2	0	0	0	0	3.0	4.7	na
125	1	1	1	0	1	0	0	3.0	18.1	0
125	2	1	1	0	1	0	1	4.0	18.1	0
125	3	1	1	0	1	0	0	4.0	15.0	0
125	4	1	2	0	1	1	0	3.0	15.0	1
126	1	1	2	0	0	1	1	2.0	18.3	0
127	1	1	1	0	1	1	0	3.0	18.1	0
128	1	1	2	0	0	3	0		5.3	1
129	1	1	1	0	0	1	0	1.0	16.0	0
129	2	1	2	0	0	1	0	3.0	4.7	0
130	1	1	2	0	0	2	0	1.5	17.5	0
131	1	1	2	1	na	na	0	1.5	na	0
131	2	1	1	0	0	3	0	1.0	4.5	0
131	3	1	1	0	0	2	0	2.0	5.3	0
131	4	1	2	0	0	1	0	2.5	8.5	0
131	5	1	1	0	0	2	0	1.0	17.5	0
131	6	1	2	0	0	1	0	3.0	1.1	0
131	7	1	1	0	0	1	0	4.0	8.8	0
131	8	1	1	0	1	2	0	4.0	13.3	1
131	9	1	2	0	1	1	0	4.0	3.3	1
132	1	1	1	0	0	2	0	1.0	5.3	0
132	2	1	2	0	0	2	0	1.0	16.0	0
132	3	1	2	0	0	2	0	1.5	17.5	0
132	4	1	2	0	0	1	0	3.0	9.6	0
132	5	1	1	0	1	1	0	4.0	19.2	0
132	6	1	1	0	1	1	0	3.5	13.3	0
133	1	1	2	0	0	1	0	4.0	16.0	0
134	1	1	1	0	0	1	0	4.0	15.5	0
134	2	1	1	0	0	1	0	4.0	15.5	0
134	3	1	2	0	1	1	0	4.0	19.2	0
134	4	1	2	0	1	1	0	4.0	18.6	0
134	5	1	2	0	1	1	0	4.0	13.3	0
134	6	1	1	0	1	4	0	4.0	3.3	0
135	1	1	1	0	1	1	0	4.0	15.0	0
135	2	1	1	0	1	1	0	4.0	6.6	0
136	1	1	1	0	1	2	0	4.0	4.4	0
137	1	1	2	0	1	1	0	4.0	4.4	1
138	1	1	0	1	na	na	0	2.0	na	0
138	2	1	1	0	0	5	1	0.5	4.5	0
138	3	1	1	0	0	4	0	0.5	5.3	0
138	4	1	2	0	0	6	0	0.5	5.3	0
139	1	1	2	1	na	na	0	4.0	na	1
139	2	1	0	0	0	7	1	0.5	4.5	0
139	3	1	2	0	0	1	0	2.0	5.3	0
139	4	1	2	0	0	1	0	3.5	18.3	0

139	5	1	1	0	0	1	1	2.0	17.5	0
139	6	1	2	0	0	1	1	3.0	17.5	0
139	7	1	0	0	0	2	0	0.5	6.1	0
139	8	1	2	0	0	1	1	2.0	4.7	0
139	9	1	0	0	0	4	0	3.0	4.7	0
139	10	1	2	0	0	1	0	4.0	6.1	1
140	1	1	2	1	na	na	0	1.5	na	0
140	2	1	2	1	na	na	0	4.0	na	0
140	3	1	0	0	0	3	1	1.0	4.8	0
140	4	1	1	0	0	2	0	1.0	5.3	0
140	5	1	0	0	0	1	0	1.0	8.5	0
140	6	1	1	0	0	1	0	1.5	14.2	1
140	7	1	2	0	0	1	0	1.0	16.0	1
140	8	1	1	0	0	2	1	1.0	18.3	1
140	9	1	1	0	0	1	0	1.5	18.1	1
140	10	1	0	0	0	1	0	1.0	18.1	0
140	11	1	2	0	0	1	0	2.0	17.5	0
140	12	1	1	0	0	4	0	1.0	17.5	0
140	13	1	0	0	0	1	0	1.0	9.7	0
140	14	1	1	0	0	2	1	0.5	6.1	0
140	15	1	2	0	0	1	0	2.0	1.1	0
140	16	1	1	0	0	1	0	3.5	6.1	0
140	17	1	2	0	0	1	0	4.0	8.8	1
141	1	1	2	1	na	na	0	2.5	na	0
141	2	1	2	0	0	0	0	2.5	4.8	0
141	3	1	0	0	0	5	0	0.5	4.5	0
141	4	1	1	0	0	2	0	2.0	4.5	0
142	1	1	1	0	0	2	0	2.5	4.8	0
142	2	1	1	0	0	1	0	3.5	8.5	0
142	3	1	2	0	0	2	0	3.0	17.5	0
142	4	1	2	0	0	1	0	3.5	9.6	0
142	5	1	1	0	1	1	0	4.0	15.0	0
143	1	1	1	0	0	2	0	2.0	14.2	0
143	2	1	1	0	0	1	0	3.0	16.0	0
143	3	1	1	0	0	3	0	2.0	18.1	0
143	4	1	2	0	0	1	0	3.5	9.6	1
143	5	1	1	0	1	1	0	4.0	18.6	0
144	1	1	2	0	0	1	0	1.5	8.5	0
144	2	1	2	0	0	1	0	1.5	14.2	1
144	3	1	1	0	0	1	0	2.0	18.3	1
144	4	1	2	0	0	1	0	1.5	17.5	1
144	5	1	2	0	0	1	0	1.0	9.7	1
144	6	1	2	0	0	1	0	2.0	4.7	1
144	7	1	0	0	0	4	1	0.8	1.1	1
144	8	1	2	0	0	1	1	1.0	1.1	1
144	9	1	2	0	0	1	0	3.0	6.1	1
144	10	1	2	0	0	1	0	4.0	8.8	1

144	11	1	2	0	0	1	0	4.0	15.5	1
144	12	1	2	0	0	1	0	4.0	19.2	1
145	1	1	1	0	0	3	1	2.0	4.8	0
145	2	1	2	0	0	5	0	1.5	4.5	0
145	3	1	2	0	0	2	0	2.0	4.5	0
145	4	1	2	0	0	2	0	1.5	4.5	0
145	5	1	2	0	0	2	0	2.0	5.3	0
145	6	1	2	0	0	1	0	2.0	8.5	0
145	7	1	2	0	0	1	0	2.5	8.5	0
145	8	1	2	0	0	1	0	2.0	14.2	0
145	9	1	2	0	0	1	0	2.0	16.0	0
145	10	1	1	0	0	1	0	1.0	18.3	0
145	11	1	2	0	0	1	0	2.0	18.1	0
145	12	1	2	0	0	1	1	2.0	9.7	0
145	13	1	2	0	0	1	1	2.0	6.1	0
145	14	1	0	0	0	2	1	2.0	1.1	0
145	15	1	2	0	0	1	1	3.0	6.1	0
145	16	1	1	0	0	7	1	4.0	9.6	0
145	17	1	2	0	0	1	0	4.0	15.5	0
145	18	1	2	0	1	1	0	4.0	19.2	0
145	19	1	1	0	1	1	1	4.0	18.1	0
145	20	1	1	0	1	1	0	4.0	15.0	0
145	21	1	1	0	1	1	0	4.0	13.3	0
145	22	1	2	0	1	1	0	4.0	4.4	0
146	1	1	1	1	na	na	0	1.5	na	0
146	2	1	2	0	0	1	0	2.0	5.3	1
147	1	1	1	0	0	1	0	4.0	18.3	0
148	1	1	2	0	0	2	0	3.0	6.1	1
148	2	1	2	0	0	2	0	4.0	6.1	1
148	3	1	2	0	0	2	0	3.0	4.7	1
148	4	1	2	0	0	1	0	4.0	1.1	1
149	1	1	2	0	0	3	0	2.5	4.7	1
149	2	1	2	0	0	2	0	2.0	1.1	1
149	3	1	2	0	0	1	0	4.0	6.1	1
150	1	1	2	0	0	1	1	1.5	1.1	0
151	1	1	1	0	0	1	0	3.5	9.6	0
152	1	1	1	0	0	2	0	2.5	15.5	1
153	1	1	2	0	1	1	0	4.0	15.0	0
153	2	1	1	0	1	4	1	4.0	13.3	0
153	3	1	1	0	1	1	0	4.0	6.8	1
154	1	1	1	0	0	2	0	3.0	4.8	1
154	2	1	1	0	0	1	0	2.0	8.5	0
154	3	1	2	0	0	2	0	3.0	17.5	0
154	4	1	2	0	0	2	1	1.5	6.1	0
154	5	1	1	0	0	2	1	2.0	4.7	1
154	6	1	1	0	0	2	1	2.0	6.1	0
154	7	1	2	0	0	2	1	4.0	9.6	0

154	8	1	2	0	1	2	0	4.0	18.1	1
154	9	1	1	0	1	2	0	4.0	6.6	1
155	1	1	2	0	0	2	0	2.0	4.5	0
155	2	1	1	0	0	2	0	3.0	8.5	0
155	3	1	2	0	0	2	0	3.0	18.1	0
155	4	1	2	0	0	3	0	2.0	6.1	0
155	5	1	2	0	0	2	0	2.5	6.1	0
155	6	1	2	0	0	2	0	4.0	15.5	0
155	7	1	1	0	1	2	0	4.0	18.1	0
155	8	1	1	0	1	2	0	4.0	4.4	0
156	1	1	2	0	0	1	0	2.0	6.1	1
156	2	1	2	0	0	2	1	4.0	8.8	0
156	3	1	2	0	1	2	0	4.0	18.1	0
156	4	1	1	0	1	3	0	4.0	13.3	0
157	1	1	1	0	0	2	1	2.5	9.6	1
157	2	1	2	0	0	2	0	4.0	9.6	0
157	3	1	2	0	0	3	0	4.0	8.8	0
157	4	1	2	0	0	1	0	4.0	15.5	0
157	5	1	1	0	1	2	0	4.0	18.1	0
158	1	1	1	0	1	2	1	4.0	18.1	1
158	2	1	1	0	1	2	0	4.0	18.1	0
159	1	1	2	0	1	1	1	4.0	15.0	1
159	2	1	2	0	1	2	0	3.5	4.4	1
160	1	1	2	0	0	2	0	4.0	4.7	1
161	1	1	2	0	0	0	0	2.0	15.5	1
162	1	1	1	0	1	1	1	4.0	15.0	0
163	1	1	2	1	na	na	0	2.0	na	0
163	2	1	2	1	na	na	0	2.5	na	0
163	3	1	2	1	na	na	0	3.0	na	0
164	1	1	2	0	0	1	0	3.0	5.3	0
164	2	1	2	0	0	2	1	2.0	14.2	0
164	3	1	1	0	0	2	0	1.0	6.1	0
165	1	1	0	0	0	1	0	3.0	5.3	0
166	1	1	2	0	0	1	1	3.0	14.2	0
166	2	1	1	0	0	1	1	3.5	18.3	0
167	1	1	1	0	0	1	0	1.0	16.0	1
168	1	1	1	0	0	2	0	2.0	16.0	0
168	2	1	2	0	0	7	1	1.0	6.1	0
168	3	1	2	0	0	2	0	3.0	15.5	0
168	4	1	1	0	1	1	0	4.0	18.1	0
169	1	1	2	0	0	1	1	2.0	18.3	0
169	2	1	1	0	0	1	0	1.0	9.7	0
169	3	1	0	0	0	1	1	3.0	6.1	0
169	4	1	2	0	0	1	0	3.5	15.5	0
169	5	1	1	0	1	2	1	4.0	18.1	0
169	6	1	1	0	1	3	0	2.5	13.3	1
170	1	1	2	0	0	1	0	3.0	1.1	0

171	1	1	1	0	0	4	1	2.5	15.5	1
172	1	1	1	0	1	2	0	4.0	18.6	0
172	2	1	1	0	1	1	0	4.0	18.1	0
173	1	1	2	1	na	na	0	3.5	na	0
173	2	1	2	1	na	na	0	2.5	na	0
173	3	1	2	1	na	na	0	3.5	na	0
173	4	1	2	0	0	1	0	0.5	5.3	0
173	5	1	1	0	0	5	0	0.5	16.0	0
173	6	1	2	0	0	1	1	2.0	17.5	0
173	7	1	2	0	0	2	0	3.0	4.7	0
173	8	1	2	0	0	2	0	3.5	9.6	0
173	9	1	1	0	1	6	1	4.0	15.0	0
173	10	1	1	0	1	1	0	4.0	4.4	0
174	1	1	2	0	1	2	0	4.0	19.2	1
174	2	1	2	0	1	1	0	4.0	18.1	1
174	3	1	2	0	1	1	0	4.0	13.3	1
175	1	1	1	0	1	1	0	3.5	13.3	1
175	2	1	0	0	1	2	1	4.0	4.4	1
176	1	1	1	0	1	1	0	4.0	15.0	1
177	1	1	1	0	1	2	0	4.0	13.3	0
177	2	1	1	0	1	1	1	3.5	4.4	0
178	1	1	1	0	1	2	0	4.0	4.4	0
179	1	1	2	1	na	na	0	3.0	na	0
179	2	1	1	0	0	1	1	2.0	4.5	0
179	3	1	2	0	0	1	0	2.0	14.2	0
179	4	1	1	0	0	5	1	1.5	18.3	0
179	5	1	1	0	0	4	1	1.0	9.7	0
179	6	1	1	0	0	1	0	1.0	1.1	0
179	7	1	1	0	0	1	0	4.0	8.8	0
179	8	1	1	0	1	4	1	3.0	18.6	0
179	9	1	1	0	1	1	0	4.0	13.3	0
180	1	1	2	0	0	0	0	3.5	4.8	0
180	2	1	1	0	0	1	0	3.0	5.3	0
180	3	1	2	0	0	1	0	0.5	16.0	0
180	4	1	0	0	0	4	1	1.0	17.5	0
180	5	1	2	0	0	2	0	2.5	4.7	0
180	6	1	1	0	0	5	1	3.5	9.6	0
180	7	1	1	0	1	2	0	4.0	19.2	0
180	8	1	2	0	1	2	0	4.0	15.0	1
180	9	1	2	0	1	1	0	4.0	13.3	1
181	1	1	2	0	0	0	0	3.0	4.8	1
181	2	1	2	0	0	1	1	3.0	4.5	0
182	1	1	2	0	0	1	0	2.0	4.8	0
182	2	1	1	0	0	1	0	1.0	8.5	1
182	3	1	2	0	0	2	0	2.5	18.3	0
183	1	1	2	0	0	1	0	1.5	4.5	0
183	2	1	0	0	0	1	0	1.0	8.5	0

183	3	1	1	0	0	1	0	2.0	18.1	1
183	4	1	0	0	0	4	1	1.0	9.7	0
183	5	1	2	0	0	2	0	2.0	4.7	1
183	6	1	2	0	0	4	0	4.0	6.1	0
183	7	1	1	0	1	1	0	4.0	18.6	1
183	8	1	1	0	1	1	0	4.0	15.0	1
183	9	1	1	0	1	2	0	4.0	6.8	1
183	10	1	2	0	1	6	1	4.0	3.3	1
184	1	1	1	0	0	1	0	2.0	4.5	0
185	1	1	2	0	0	1	0	3.5	4.5	1
186	1	1	1	0	1	1	0	4.0	18.1	0
187	1	1	1	0	1	2	0	4.0	15.0	0
187	2	1	1	0	1	2	0	4.0	6.8	0
188	1	1	1	0	0	4	0	2.0	6.1	0
189	1	1	1	0	1	1	0	2.0	18.1	0
190	1	1	2	1	na	na	0	2.0	na	1
190	2	1	2	1	na	na	0	2.0	na	1
190	3	1	2	1	na	na	0	2.0	na	1
190	4	1	2	1	na	na	0	2.0	na	1
190	5	1	1	1	na	na	0	1.0	na	0
190	6	1	1	1	na	na	0	1.0	na	0
190	7	1	2	1	na	na	0	1.0	na	1
190	8	1	1	1	na	na	0	1.0	na	0
190	9	1	2	1	na	na	0	1.0	na	1
190	10	1	1	1	na	na	0	1.0	na	1
190	11	1	1	1	na	na	0	1.0	na	1
190	12	1	2	1	na	na	0	1.0	na	0
190	13	1	1	1	na	na	0	1.0	na	0
190	14	1	2	1	na	na	0	1.0	na	0
190	15	1	1	1	na	na	0	1.0	na	0
190	16	1	2	1	na	na	0	1.0	na	0
190	17	1	1	1	na	na	0	1.0	na	0
190	18	1	2	1	na	na	0	3.0	na	0
190	19	1	2	1	na	na	0	2.5	na	0
190	20	1	2	1	na	na	0	3.0	na	1
190	21	1	2	1	na	na	0	4.0	na	1
190	22	1	2	1	na	na	0	4.0	na	1
190	23	1	2	1	na	na	0	4.0	na	1
190	24	1	2	1	na	na	0	4.0	na	1
190	25	1	2	1	na	na	0	4.0	na	1
191	1	1	1	1	na	na	0	1.0	na	1
191	2	1	1	1	na	na	0	1.0	na	0
191	3	1	1	1	na	na	0	1.0	na	0
191	4	1	1	1	na	na	1	3.0	na	0
191	5	1	1	1	na	na	0	3.5	na	0
191	6	1	2	1	na	na	0	3.5	na	0
191	7	1	2	1	na	na	0	4.0	na	0

191	8	1	1	1	na	na	0	3.5	na	0
191	9	1	1	1	na	na	0	3.5	na	1
191	10	1	1	1	na	na	0	4.0	na	0
191	11	1	1	1	na	na	0	3.5	na	0
191	12	1	1	1	na	na	0	2.5	na	1
191	13	1	2	1	na	na	1	3.0	na	1
192	1	1	1	1	na	na	0	1.0	na	1
192	2	1	1	1	na	na	0	2.0	na	1
192	3	1	1	1	na	na	0	2.0	na	1
192	4	1	2	1	na	na	0	2.0	na	1
192	5	1	2	1	na	na	0	2.0	na	1
193	1	1	2	1	na	na	0	1.0	na	0
193	2	1	1	1	na	na	0	2.0	na	0
193	3	1	1	1	na	na	0	2.0	na	0
193	4	1	1	1	na	na	0	2.0	na	0
193	5	1	1	1	na	na	0	2.0	na	0
193	6	1	1	1	na	na	0	2.0	na	0
193	7	1	1	1	na	na	0	2.0	na	0
193	8	1	1	1	na	na	0	2.0	na	0
193	9	1	1	1	na	na	0	4.0	na	0
193	10	1	2	1	na	na	0	4.0	na	1
193	11	1	2	1	na	na	0	4.0	na	0
193	12	1	2	1	na	na	0	4.0	na	1
193	13	1	2	1	na	na	0	4.0	na	1
194	1	1	0	1	na	na	0	1.5	na	0
194	2	1	1	1	na	na	0	3.0	na	0
194	3	1	1	1	na	na	0	3.0	na	1
194	4	1	1	1	na	na	0	1.5	na	0
194	5	1	1	1	na	na	0	1.5	na	1
194	6	1	1	1	na	na	0	2.0	na	1
194	7	1	1	1	na	na	0	1.5	na	1
195	1	1	2	0	0	0	0	4.0	20.0	0
196	1	1	1	0	0	0	0	4.0	20.0	1
196	2	1	1	0	0	0	1	4.0	20.0	1
196	3	1	2	0	1	0	0	3.5	20.0	1
197	1	1	2	0	0	0	0	4.0	20.0	1
197	2	1	1	0	0	0	0	3.5	20.0	0
197	3	1	2	0	0	1	0	2.0	8.8	0
197	4	1	1	0	1	7	0	4.0	18.1	0
197	5	1	1	0	1	0	0	4.0	20.0	1
198	1	1	2	0	0	0	0	4.0	20.0	0
199	1	1	1	0	0	0	0	3.0	20.0	0
199	2	1	2	0	0	0	0	4.0	20.0	0
199	3	1	2	0	0	8	0	4.0	8.8	1
199	4	1	1	0	0	0	0	4.0	20.0	1
200	1	1	2	0	0	0	0	4.5	20.0	0
200	2	1	2	0	0	0			20.0	0

200	3	1	2	0	0	0	0	4.0	20.0	0
201	1	1	1	0	0	0	0	3.0	20.0	0
201	2	1	2	0	0	0	0	4.0	20.0	0
201	3	1	2	0	0	0	0	3.0	20.0	1
202	1	1	1	0	0	0	0	3.0	20.0	0
202	2	1	1	0	0	0	0	3.0	20.0	0
203	1	1	2	0	0	9	0	4.0	20.0	0
203	2	1	1	0	0	3	0	3.0	20.0	0
203	3	1	2	0	0	7	1	3.0	9.6	0
203	4	1	2	0	1	5	1	4.0	18.6	0
203	5	1	2	0	1	1	0	4.0	6.6	1
204	1	1	2	0	0	0	0	4.0	20.0	0
204	2	1	1	0	1	0	0	4.0	20.0	0
205	1	1	2	0	0	1	0	3.0	4.7	0
205	2	1	2	0	0	1	0	3.5	9.6	0
205	3	1	2	0	1	0	0	4.0	18.6	0
206	1	1	2	0	0	0	0	3.0	4.7	0
206	2	1	2	0	0	1	0	3.5	9.6	0
207	1	1	2	0	0	1	1	3.0	6.1	0
207	2	1	1	0	0	0	1	2.0	15.5	0
208	1	1	1	0	0	2	0		14.2	0
208	2	1	2	0	0	1	1	3.0	17.5	1
209	1	1	2	0	0	0	0	4.0	14.2	0
209	2	1	2	0	0	1	0	2.5	9.7	0
209	3	1	2	0	0	1	0	2.0	1.1	0
209	4	1	2	0	0	1	0	3.5	15.5	0
209	5	1	1	0	1	9	0	4.0	18.1	0
209	6	1	0	0	1	1	0	3.5	13.3	0
210	1	1	2	0	0	1	1	1.5	6.1	0
211	1	1	2	0	0	1	0	3.5	18.3	1
212	1	1	1	0	0	0	0	3.5	18.3	0
212	2	1	2	0	0	0	0	4.0	9.7	0
212	3	1	1	0	0	0	0	3.0	1.1	0
212	4	1	2	0	0		1	3.5	8.8	0
212	5	1	2	0	1	3	0	2.0	18.1	0
213	1	1	2	0	0	0	0	4.0	17.5	0
213	2	1	0	0	0	0	0	4.0	1.1	0
213	3	1	1	0	0	1	0	2.0	8.8	0
213	4	1	1	0	0	3	1	2.5	15.0	0
214	1	1	0	0	0	5	1	2.0	1.1	0
215	1	1	1	0	0	3	0	2.0	1.1	0
215	2	1	2	0	0	1	0	4.5	8.8	0
216	1	1	1	0	0	2	0	1.0	8.5	0
216	2	1	0	0	0	2	0	1.0	18.3	0
216	3	1	2	0	0	1	0	3.0	9.7	0
216	4	1	1	0	0	1	0	2.5	1.1	0
216	5	1	2	0	0	1	1	4.0	15.5	0

216	6	1	1	0	1	1	0	4.0	18.1	0
216	7	1	1	0	1	1	0	4.0	6.6	0
217	1	1	1	0	0	1	1		14.2	0
217	2	1	2	0	0	0	1	3.5	18.3	0
217	3	1	1	0	0	1	1	1.0	9.7	0
217	4	1	1	0	0	1	0	2.0	1.1	0
217	5	1	1	0	0	1	0	4.0	8.8	0
217	6	1	2	0	1	1	1	3.0	18.6	1
218	1	1	1	0	0	1	0	2.0	9.7	1
219	1	1	2	0	0	1	0	2.5	16.0	0
219	2	1	1	0	0	1	0	3.0	18.1	0
220	1	1	0	0	0	2	0	2.0	5.3	0
220	2	1	1	0	0	1	0		14.2	0
220	3	1	1	0	0	4	0	1.0	16.0	1
220	4	1	0	0	0	5	0	1.5	17.5	0
220	5	1	2	0	0	2	0	2.0	9.6	0
220	6	1	2	0	1	4	0	2.0	19.2	0
221	1	1	1	0	0	3	0	1.0	16.0	1
221	2	1	0	0	0	2	0	1.0	18.1	0
221	3	1	0	0	0	2	0	1.0	9.7	0
221	4	1	0	0	0	3	1	1.0	1.1	0
221	5	1	0	0	0	2	0	0.5	8.8	0
222	1	1	2	0	0	7	1	0.5	14.2	0
222	2	1	1	0	0	4	0	1.0	18.3	0
222	3	1	0	0	0	4	1	0.5	17.5	0
222	4	1	1	0	0	3	1	1.0	6.1	0
222	5	1	0	0	0	2	0	1.0	1.1	0
222	6	1	0	0	0	2	1	0.5	9.6	0
222	7	1	2	0	1	6	1	2.0	18.6	0
223	1	1	1	0	0	1	0	2.0	14.2	1
224	1	1	2	0	1	0	0	4.0	13.3	0
225	1	1	1	0	1	0	0	2.5	13.3	1
226	1	1	2	0	0	4	0	1.5	8.5	0
226	2	1	2	0	0	4	0	4.0	17.5	0
226	3	1	2	0	0	1	0	3.0	8.8	0
227	1	1	1	0	0	2	0	2.5	16.0	0
228	1	1	2	0	0	3	1	2.0	18.3	0
229	1	1	2	0	0	3	1	1.5	17.5	0
230	1	1	2	0	0	11	0	3.0	9.7	0
231	1	1	2	0	0	1	0	3.0	17.5	1
232	1	1	2	0	0	0	0	4.0	9.6	1
233	1	1	2	0	0	4	1	3.5	1.1	1
234	1	1	2	0	1		0	4.0	18.1	0

Average Temp: average temperature per month; CP: centrifugation protocol, 0 = one-step, low speed, 1 = two-step, high speed; *EGFR* mut ctDNA: *EGFR* mutated ctDNA, 0 = not detected, 1 = detected; *EGFR* status: *EGFR* mutational status as determined by tissue and/or liquid biopsy, 0 = *EGFR* WT, 1 = *EGFR* mutated; Hemolysis: 0 = no visible hemolysis 1 = visible hemolysis present; Na: not applicable; Sens: reached assay sensitivity (maximal detectable *EGFR* mutation frequency), 0 >3%, 1 = 0.5 – 3%, 2 <0.5%; Transit Time:

number of days between sample collection and processing; Tube: 0 = Streck tube, 1 = EDTA tube; # mL: volume of generated plasma.
